# Prediction of type 2 diabetes mellitus using hematological factors based on machine learning approaches: a cohort study analysis

**DOI:** 10.1038/s41598-022-27340-2

**Published:** 2023-01-12

**Authors:** Amin Mansoori, Toktam Sahranavard, Zeinab Sadat Hosseini, Sara Saffar Soflaei, Negar Emrani, Eisa Nazar, Melika Gharizadeh, Zahra Khorasanchi, Sohrab Effati, Mark Ghamsary, Gordon Ferns, Habibollah Esmaily, Majid Ghayour Mobarhan

**Affiliations:** 1 International UNESCO Center for Health-Related Basic Sciences and Human Nutrition, Mashhad University of Medical Sciences, Mashhad, 99199-91766 Iran; 2 Department of Applied Mathematics, Ferdowsi University of Mashhad, Mashhad, Iran; 3 Faculty of Medicine, Islamic Azad University of Mashhad, Mashhad, Iran; 4 Student Research Committee, School of Medicine, Mashhad University of Medical Science, Mashhad, Iran; 5 Student Research Committee, Department of Biostatistics, School of Health, Mashhad University of Medical Sciences, Mashhad, Iran; 6 Student Research Committee, School of Paramedical Sciences, Mashhad University of Medical Sciences, Mashhad, Iran; 7 Department of Nutrition, School of Medicine, Mashhad University of Medical Sciences, Mashhad, Iran; 8 School of Public Health, Loma Linda University, Loma Linda, CA USA; 9 Division of Medical Education, Brighton and Sussex Medical School, Brighton, UK; 10 Social Determinants of Health Research Center, Mashhad University of Medical Sciences, Mashhad, Iran; 11 Department of Biostatistics, School of Health, Mashhad University of Medical Sciences, Mashhad, Iran

**Keywords:** Metabolic disorders, Machine learning, Statistical methods, Predictive markers

## Abstract

Type 2 Diabetes Mellitus (T2DM) is a significant public health problem globally. The diagnosis and management of diabetes are critical to reduce the diabetes complications including cardiovascular disease and cancer. This study was designed to assess the potential association between T2DM and routinely measured hematological parameters. This study was a subsample of 9000 adults aged 35–65 years recruited as part of Mashhad stroke and heart atherosclerotic disorder (MASHAD) cohort study. Machine learning techniques including logistic regression (LR), decision tree (DT) and bootstrap forest (BF) algorithms were applied to analyze data. All data analyses were performed using SPSS version 22 and SAS JMP Pro version 13 at a significant level of 0.05. Based on the performance indices, the BF model gave high accuracy, precision, specificity, and AUC. Previous studies suggested the positive relationship of triglyceride-glucose (TyG) index with T2DM, so we considered the association of TyG index with hematological factors. We found this association was aligned with their results regarding T2DM, except MCHC. The most effective factors in the BF model were age and WBC (white blood cell). The BF model represented a better performance to predict T2DM. Our model provides valuable information to predict T2DM like age and WBC.

## Introduction

Diabetes is a metabolic disease that shows itself clinically as chronic hyperglycemia, blood lipid and protein abnormalities, and other symptoms that increase the risk of morbidity and mortality^1^. Diabetes is a significant public health issue in the U.S. and around the world; it has been categorized as type 1, type 2, and gestational diabetes^2^. Type 2 diabetes mellitus (T2DM) is rising is relation to urbanization, population aging, and related lifestyle changes, especially in people over 65^3^. Adults with diabetes were anticipated to number 415 million worldwide in 2015, and by 2040, that number will increase to 642 million^2,4^. The national Coronary Artery Disease (CAD) risk factors monitoring report estimates that among Iranians aged 15–64, the prevalence of diabetes was 8.7% in overall, with nearly half (4.1%) of those patients were newly diagnosed cases^5^. Diabetes is a serious and chronic disorder that has a significant negative impact on people's lives, families, and societies all over the world. Uncontrolled diabetes also increases the risk of metabolic, cellular, and blood disturbances leading to vascular complications, cancer, and all-cause death. According to estimates, it was one of the top 10 causes of mortality for adults and resulted in 4 million deaths worldwide in 2017^2,4^. Despite the lack of the typical hematologic pathologic features associated with T2DM, several hematologic abnormalities have been identified in patients with this illness. It has been demonstrated that T2DM is closely related with several hematological abnormalities affecting platelets (PLTs), white blood cells (WBCs), red blood cells (RBCs), and the coagulation systems^6,7^.

Recent studies have indicated a correlation between some hematological parameters and diabetes, such as a reduction in RBC count in developing T2DM, and an increase in total WBC and PLT count in type 2 diabetic patients^7–9^. However, previous studies identified no relationship between T2DM and the other hematological parameters. Although a cross-sectional study has shown the association between the hematological parameters and T2DM in adult patients, its sample size was meaningfully lower than the current study^7^.

An early diagnosis and management of diabetes are crucial to reducing the risks of cardiovascular disease, cancer, and mortality due to the rising prevalence of diabetes and its relation to these diseases. The objective of this present study was to determine the association between diabetes and hematological factors.

## Methods

### Participants

The participants were recruited from the baseline of the Mashhad Stroke and Heart Atherosclerotic Disorders (MASHAD) study, Mashhad, north-eastern Iran, following a similar research protocol^10^. Nine thousand seven hundred four (9704) individuals aged 35–65 years were enrolled regarding their T2DM status were studied from the baseline of this cohort. T2DM was defined as a fasting blood glucose (FBG) ≥ 126 mg/dl or being treated with available oral hypoglycemic medications or insulin. Also, we consider triglyceride-glucose (TyG) index for the diagnosis of T2DM that defined as follows^11^:

$$TyG = \ln \left( {\frac{{\text{triglyceride* glucose }}}{2}} \right)$$

**Figure 1:**
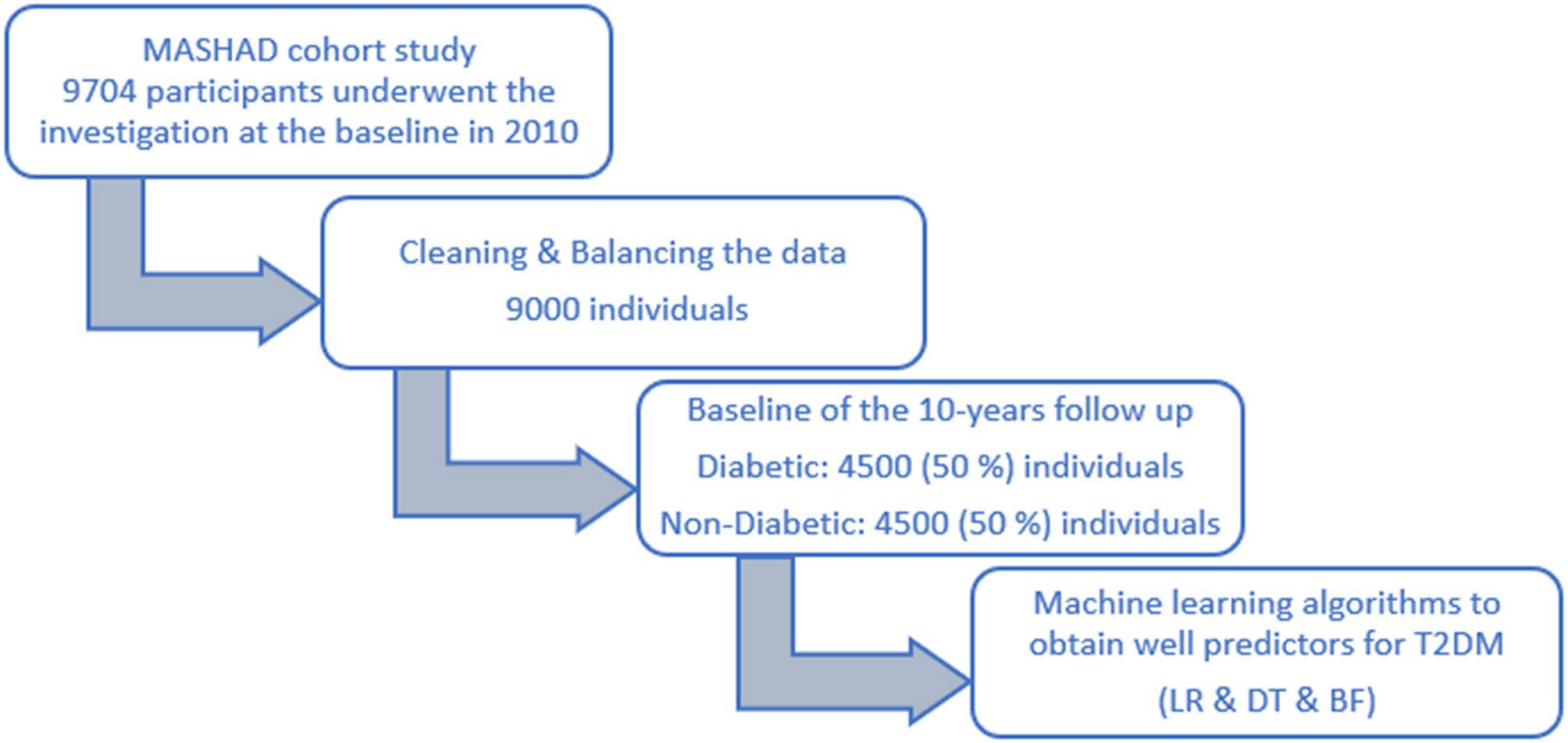
Flow chart of this study.

Also, we categorize the TyG index by using the median of our data. The median of TyG index in our data is 8.831. The inclusion criteria were males and females between the age of 35 and 65 years. We are dealing with data that is unbalanced (Diabetic vs. Non-Diabetic) in this investigation. One of the approaches that can be used for solving this problem is Synthetic Minority Oversampling Technique (SMOTE)^12^. The SMOTE algorithm is one of the most widely used under sampling and over sampling methods that create synthetic minority class samples. Therefore, in this study, the SMOTE algorithm was used to balance the classes. The observations were then analyzed on a balanced data set and after cleaning the data in each of the measured variables, finally with 9000 observations. After the cleaning data, we used the data from 9000 individuals in this study (Fig. 1).

At the beginning of this study, we measured the demographic characteristics (including gender and age) and hematological information including HGB (Hemoglobin), HCT (Hematocrit), MCH (Mean Corpuscular Hemoglobin), PLT (Platelet count), LYM (Lymphocyte Count), MXD (Mixed Cell Count), NEUT (Neutrophil Count), RDW (Red cell Distribution Width), PDW (Platelet Distribution Width), MPV (Mean Platelet Volume), RBC (Red Blood Cell), MCV (Mean Corpuscular Volume), MCHC (Mean Corpuscular Hemoglobin Concentration), and WBC (White Blood Cell).

### Blood sampling

According to a standard protocol, all blood samples were taken from an antecubital vein of all participants who were in a sitting position, between 8–10 am, after 14 h of fasting. The samples were collected in 20 ml vacuum tubes and centrifuged for 30–45 min to separate the serum and plasma, and later sent to Bu Ali Research Institute, Mashhad, for laboratory examinations. Aliquots of serum were also kept frozen at -80 C for future analysis. The details of laboratory measurements and cut-offs are explained in the baseline report of the MASHAD cohort study^10^.

### Statistical analysis and model building

To describe the quantitative and qualitative variables, mean ± SD and frequency (%) were reported, respectively. Chi-square and Fisher’s exact tests were applied to measure the association between categorical variables. Also, the mean of quantitative variables between the two groups were compared by independent T test. In addition, machine learning techniques such as logistic regression (LR) and decision tree (DT) algorithms have been used to analyze data. In fact, we applied these algorithms to deduce the association between T2DM and hematological factors. We considered two models for the prediction of T2DM. Model I investigated the association of T2DM with hematological factors and Model II investigated the association of the TyG index with hematological factors. All analysis were performed using SPSS version 22 (Armonk, NY: IBM Corp.) and SAS JMP Pro (SAS Institute Inc., Cary, NC) at the significant level of 0.05.

### Logistic regression (LR) modeling

Logistic Regression is a popular model to evaluate the relationship between various predictor variables (either categorical or continuous) and binary outcomes in medicine, public health, etc.^13^.

Let ${Y}_{i}$ denotes the response variable and takes the values of 0 or 1 depending on whether response occurs or not. Also, ${\varvec{X}}$ be vectors of covariates associated with response variable, ${\varvec{\beta}}$ is the corresponding vectors of regression coefficients. So, the association between the covariates and binary response variable can be investigated as follows:

$$logit\left\{ {E\left( {Y_{i} } \right)} \right\} = logit\{ Pr(Y_{i} = 1| {\varvec{X}},{\varvec{\beta}})\} = {\varvec{\beta}}^{{\varvec{T}}} {\varvec{X}}.$$

### Decision tree (DT) modelling

Machine learning is one of the artificial intelligence analyses that emerged in the late twentieth century^14,15^. In other words, machine learning is a process for extracting hidden knowledge in large data sets. One of the important problems for researchers in this process is data classification^16^. There are different techniques for classification problems^16^. DT can be applied in various applications in the medical field^17–19^. Due to the simplicity in understanding and clarity and extracting simple and understandable rules, it is widely applied and studied in these fields^16^. The DT consists of components, nodes, and branches. So that, there are three types of nodes: (1) a root node represents the result of subdividing all records into two or more exclusive subsets. (2) The internal nodes represent a possible point in the tree structure connected to the root node from the top and the leaf nodes from the bottom. (3) Leaf nodes that show the tree’s final results in dividing records into target groups. Branches in the tree indicate the chance of placing records in target groups that emanate from the root node and the internal nodes^14,15^. DT algorithm uses the Gini impurity index for selecting the best variable.

$$Gini\left( D \right) = 1 - \mathop \sum \limits_{i = 1}^{m} P_{i}^{2}$$

where ${P}_{i}$ is the probability that a record in D belongs to the class ${C}_{i}$ and is estimated by |${C}_{i}$,D|/|D. Logistic regression or LR is a statistical model applied to modeling dichotomous targets and investigating the effect of explanatory variables on the dichotomous target variable. In LR, the probability of placing each of the records in the target groups is also presented^20,21^. The main advantage of using the LR is that it can provide a good direct or inverse relationship between the inputs or explanatory variables and the target. It is also a flexible method^22^.

### Bootstrap forest (BF) modeling

BF platform fits an ensemble model by averaging several decision trees, each of which is fit to a bootstrap sample of the training data. Each split in each tree shows a random subset of the predictors. In this way, many weak models are combined to produce a stronger model. The final prediction for an observation is the average of the predicted values for that observation over all the decision trees. In fact, the BF determines the significant factors associated with diabetes.

Receiver operating characteristic (ROC) curves were used to evaluate the accuracy, precision, and specificity for all three algorithms. Also, the confusion matrix of the three algorithms were given.

### Ethics approval

All the participants consented to take part in the study by signing written informed consent. The study protocol was reviewed and all methods are approved by the Ethics Committee of Mashhad University of Medical Sciences with approval number IR.MUMS.REC.1399.660. All methods were carried out in accordance with relevant guidelines and regulations.

## Results

A total of 9000 complete datasets of participant were analyzed in this cohort study (N = 4500 with Diabetes [female 62.77% vs male 37.22%], N = 4500 without Diabetes [female 59.15% vs male 40.84%]). The main baseline characteristics of the study population are summarized in Table 1. All the variables were significantly different between the two groups, including age, WBC, PDW, RDW, RBC, sex, PLT, MCHC, and HCT (*P* < 0.05). According to previous studies on the positive relationship of the TyG index with the presence of T2DM, we also considered the association of the TyG index with the hematological factors^11,23,24^.

**Table 1:** Clinical characteristics at the baseline of Mashhad stroke and heart atherosclerotic disorder (MASHAD) study used in this paper.

Variable	Model I			Model II		
	Diabetic (n = 4500)	Non-Diabetic (n = 4500)	P***	TyG ≥ 8.831 (n = 4489)	TyG < 8.831 (n = 4489)	P***
Age (year)	52.18 ± 7.54*	48.00 ± 8.17	< 0.001	51.85 ± 7.52	48.34 ± 8.36	
Sex (n)			0.004	1727 (38.47%) 2762 (61.52%)	1771 (39.45%) 2718 (60.54%)	
Male	1675 (37.22%)**	1838 (40.84%)				
Female	2825 (62.77%)	2662 (59.15%)				
HGB (g/dl)	13.81 ± 1.50	13.75 ± 1.95	0.001	13.93 ± 1.90	13.64 ± 1.55	
HCT (%)	41.28 ± 3.70	41.17 ± 3.97	0.004	41.48 ± 3.70	40.98 ± 3.95	
MCH (pg)	28.12 ± 2.49	28.43 ± 2.47	< 0.001	28.21 ± 2.42	28.34 ± 2.54	
PLT (× 10^3^/µl)	237.82 ± 66.46	227.97 ± 58.34	0.001	238 ± 67.38	226.69 ± 56.99	
LYM (%)	2.61 ± 4.96	2.16 ± 0.89	0.001	2.65 ± 4.97	2.13 ± 0.89	
MXD (%)	0.66 ± 0.55	0.62 ± 0.31	0.002	0.67 ± 0.55	0.61 ± 0.31	
NEUT (%)	3.63 ± 1.37	3.30 ± 3.42	< 0.001	3.68 ± 3.47	3.25 ± 1.17	
RDW (%)	40.98 ± 3.06	41.90 ± 3.19	< 0.001	40.90 ± 3.12	41.97 ± 3.11	
PDW (%)	12.94 ± 2.04	12.68 ± 1.96	0.011	12.88 ± 2.01	12.47 ± 2.00	
MPV (fl)	10.05 ± 0.95	10.01 ± 0.93	0.360	10.02 ± 0.92	10.05 ± 0.96	
RBC (× 10^6^/µl)	4.92 ± 0.48	4.84 ± 0.48	< 0.001	4.94 ± 0.49	4.82 ± 0.47	
MCV (fl)	83.99 ± 5.84	85.02 ± 6.10	< 0.001	84.09 ± 5.58	84.90 ± 6.36	
MCHC (g/dl)	33.43 ± 1.47	33.32 ± 1.52	0.001	33.49 ± 1.56	33.26 ± 1.42	
WBC (× 10^3^/µl)	6.62 ± 1.63	6.01 ± 1.53	< 0.001	6.64 ± 1.60	5.98 ± 1.54	

*Mean ± Sd.

**n(%).

***P-value based on 2 sample t-test for Mean ± Sd and Chi-sq test for frequency (%).

*HGB* Hemoglobin, *HCT* Hematocrit, *MCH* Mean corpuscular hemoglobin, *PLT* Platelet count, *LYM* Lymphocyte count, *MXD* Mixed cell count, *NEUT* Neutrophil count, *RDW* Red cell distribution width, *PDW* Platelet Distribution Width, *MPV* Mean platelet volume, *RBC* Red blood cell, *MCV* Mean corpuscular volume, *MCHC* Mean corpuscular hemoglobin concentration, *WBC* White blood cell, *TyG* Triglyceride-glucose.

Three machine learning techniques were used to investigate the relationship between hematological predictors and binary response variables (diabetic, and non-diabetic). So, the main objective of this study was to anticipate diabetes using the LR, DT, and BF models and to determine their associated factors, especially hematological markers. For this purpose, the dataset was randomly split into two parts: training data, and test data (75% vs 25%). The training dataset was utilized to develop the DT and BF models, which was then validated using test data (25%) that hadn't been used during training.

### LR model

Results from the multiple LR model revealed that all variables were significantly associated with having of diabetes (*P* < 0.05). In other words, our findings after adjusting the effect of other variables in the Model I presented that the odds of having diabetes in males is 0.69 times than of females (*P* < 0.05). Also, after adjusting the effect of other variables for each increasing in age, the odds of having diabetes raises by 8 percent (*P* 0.05). Among the analyzed hematological variables, age (OR = 1.08, 95%CI = (1.07,1.08)), WBC (OR = 1.29, 95%CI = (1.24,1.33)), and PDW (OR = 1.11, 95%CI = (1.08,1.14)), had the greatest associations with having of diabetes, especially WBC because for each unit increase in WBC, the odds of having diabetes increases by 29 percent (P < 0.001) (Table 2 Model I). Also, our findings after adjusting the effect of other variables in the Model II presented that the odds of having high TyG index in males is 0.66 times than of females (*P* < 0.05). Also, after adjusting the effect of other variables for each increasing in age, the odds of having high TyG index raises by 7 percent (P < 0.05). Among the analyzed hematological variables, age (OR = 1.07, 95%CI = (1.06, 1.08)), RBC (OR = 1.74, 95%CI = (1.36, 1.38)), WBC (OR = 1.33, 95%CI = (1.28,1.38)), and PDW (OR = 1.08, 95%CI = (1.05,1.12)), had the greatest associations with having high TyG index, especially WBC because for each unit increase in WBC, the odds of having high TyG index increases by 33 percent (*P* < 0.001) (Table 2 Model II).

**Table 2:** The results of multiple LR model.

Variables (Ref)	Model I		Model II	
	OR^#^ (95% CI)	*P*	OR^#^ (95% CI)	*P*
WBC	1.29 (1.24, 1.33)	< 0.001	1.33 (1.28, 1.38)	< 0.001
RDW	0.92 (0.90, 0.94)	< 0.001	0.90 (0.88, 0.93)	< 0.001
**Sex(female)**				
male	0.69 (0.60, 0.79)	0.004	0.66 (0.57, 0.76)	< 0.001
MCHC	1.13 (1.07, 1.19)	0.001	0.82 (0.70, 0.96)	0.017
Age	1.08 (1.07, 1.09)	< 0.001	1.07 (1.06, 1.08)	< 0.001
RBC	2.11 (1.65, 2.69)	< 0.001	1.74 (1.36, 1.38)	< 0.001
HCT	0.93 (0.90, 0.96)	0.004	0.74 (0.65, 0.85)	< 0.001
PLT	1.002 (1.001, 1.003)	0.001	1.004 (1.003, 1.005)	< 0.001
PDW	1.11 (1.08, 1.14)	0.011	1.08 (1.05, 1.12)	< 0.001
HGB	–	–	2.25 (1.53, 3.31)	< 0.001

^#^*OR* = odds ratio

*HCT* Hematocrit, *PLT* Platelet count, *RDW* Red cell distribution width, *PDW* Platelet distribution width, *RBC* Red blood cell, *MCHC* Mean corpuscular hemoglobin concentration, *WBC* White blood cell, *HGB* Hemoglobin.

**Figure 2:**
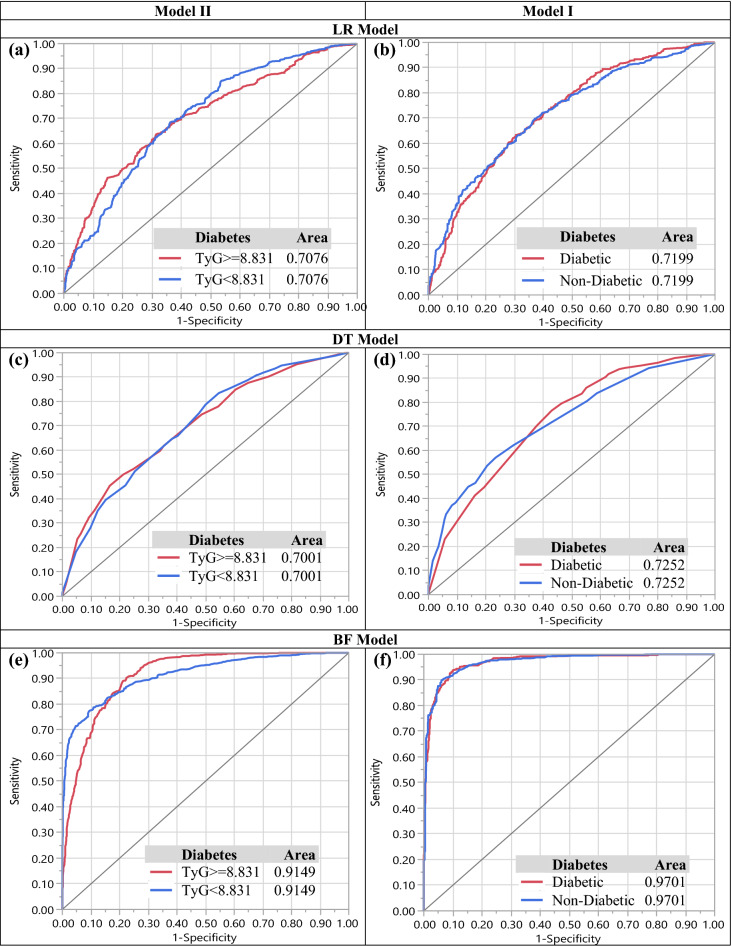
ROC curves for LR, DT, and BF algorithms for models I and II. Figures (**a**, **c** and **e**) show the ROC curves for LR, DT, and BF algorithms in model I. Also, figures (**b**, **d** and **f**) show the ROC curves for LR, DT, and BF algorithms in model II.

For comparison models the confusion matrices of the models I and II are given in Table 4. Moreover, Fig. 2 (a) and (b) depicts the ROC curves of the models I and II.

### DT model

Figures S1 and S2 in Supplementary Information file illustrates the outcomes of the DT training for hematological factors. The DT algorithm determined the various diabetes risk factors and categorized them into 5 layers. According to the DT model, the first variable (root) is of the utmost significance for classifying data, with the subsequent variables having the subsequent levels of significance^25^. Figures S1 and S2 in Supplementary Information file illustrates that WBC, followed by age and RDW, has the greatest impact on the diabetes presence risk for models I and II.

In Model I participants with age < 47, WBC < 5.9, and RDW ≥ 41.2 had lower diabetes, according to the DT model, than those with higher WBC and RDW levels and older ages (0.8793 vs. 0.1207 incident rate). Eighty percent of patients had diabetes in the subgroup with older age (> = 47), low RDW (41.7), and high WBC (> = 6.8). More diabetes cases were represented by older age, higher WBC, and lower RDW levels than their corresponding opposite groupings. Table 3 (Model I) illustrates the specific diabetic rules developed by the DT model. The important variables in Table 2 (Model I) are used as input for this model. Age and WBC were thus determined to be the most crucial variables in the DT model and in the diagnosis of diabetes. In Model II participants with age < 47, WBC < 6.1, and RDW ≥ 39.4 had lower TyG index, according to the DT model, than those with higher WBC and RDW levels and older ages (0.8341 vs. 0.1659 incident rate). Eighty three percent of patients had high TyG index in the subgroup with older age (> = 47), high WBC (> = 6.3), and low RDW (< 41.7). Cases with high TyG index were represented by older age, higher WBC, and lower RDW levels than their corresponding opposite groupings. Table 3 (Model II) illustrates the specific rules developed by the DT model. The important variables in Table 2 (Model II) are used as input for this model. Age and WBC were thus determined to be the most crucial variables in the DT model and in the diagnosis of diabetes. For evaluation, the confusion matrices of the models I and II are given in Table 4. Moreover, Fig. 2(c) and (d) depicts the ROC curves of the models I and II.

**Table 3:** Detailed rules based on DT in models I and II.

Model I		
Rules	Diabetic (%)	Non-Diabetic (%)
R1: Age < 47 & WBC < 5.9 & RDW ≥ 41.2	12.07	87.93
R2: Age < 47 & WBC < 5.9 & RDW < 41.2 & PDW < 16.3 & HCT ≥ 39.9	15.73	84.27
R3: Age < 47 & WBC < 5.9 & RDW < 41.2 & PDW < 16.3 & HCT < 39.9	35.86	64.14
R4: Age < 47 & WBC < 5.9 & RDW < 41.2 & PDW ≥ 16.3	72.47	27.53
R5: Age < 47 & WBC ≥ 5.9 & Age < 40 & PDW < 12.8	12.76	87.24
R6: Age < 47 & WBC ≥ 5.9 & Age < 40 & PDW ≥ 12.8 & Age ≥ 38	14.85	85.15
R7: Age < 47 & WBC ≥ 5.9 & Age < 40 & PDW ≥ 12.8 & Age < 38	44.05	55.95
R8: Age < 47 & WBC ≥ 5.9 & Age ≥ 40 & PLT < 249 & RDW ≥ 44.9	01.02	98.98
R9: Age < 47 & WBC ≥ 5.9 & Age ≥ 40 & PLT < 249 & RDW < 44.9	39.74	60.26
R10: Age < 47 & WBC ≥ 5.9 & Age ≥ 40 & PLT ≥ 249 & PDW < 12	47.37	52.63
R11: Age < 47 & WBC ≥ 5.9 & Age ≥ 40 & PLT ≥ 249 & PDW ≥ 12	66.73	33.27
R12: Age ≥ 47 & RDW ≥ 41.7 & WBC < 5.5 & Sex(male)	15.85	84.15
R13: Age ≥ 47 & RDW ≥ 41.7 & WBC < 5.5 & Sex(female) & Age < 50	18.00	82.00
R14: Age ≥ 47 & RDW ≥ 41.7 & WBC < 5.5 & Sex(female) & Age ≥ 50	46.30	53.70
R15: Age ≥ 47 & RDW ≥ 41.7 & WBC ≥ 5.5 & MCHC < 35.4 & MCHC ≥ 34.9	1.85	98.15
R16: Age ≥ 47 & RDW ≥ 41.7 & WBC ≥ 5.5 & MCHC < 35.4 & MCHC < 34.9	56.22	43.78
R17: Age ≥ 47 & RDW ≥ 41.7 & WBC ≥ 5.5 & MCHC ≥ 35.4	89.24	10.76
R18: Age ≥ 47 & RDW < 41.7 & WBC < 6.8 & RBC < 4.4 & Age < 52	1.37	98.63
R19: Age ≥ 47 & RDW < 41.7 & WBC < 6.8 & RBC < 4.4 & Age ≥ 52	36.75	63.25
R20: Age ≥ 47 & RDW < 41.7 & WBC < 6.8 & RBC ≥ 4.4 & PDW < 11.8	49.45	50.55
R21: Age ≥ 47 & RDW < 41.7 & WBC < 6.8 & RBC ≥ 4.4 & PDW ≥ 11.8	67.42	32.58
R22: Age ≥ 47 & RDW < 41.7 & WBC ≥ 6.8	80.89	19.11

**Model II**		
Rules	TyG < 8.831 (%)	TyG ≥ 8.831 (%)
R1: Age ≥ 47 & WBC ≥ 6.3 & RDW < 41.7 & Age ≥ 52	16.17	83.83
R2: Age ≥ 47 & WBC ≥ 6.3 & RDW < 41.7 & Age < 52	28.50	71.50
R3: Age ≥ 47 & WBC ≥ 6.3 & RDW ≥ 41.7 & MCHC ≥ 35.4	5.05	94.95
R4: Age ≥ 47 & WBC ≥ 6.3 & RDW ≥ 41.7 & MCHC < 35.4	44.08	55.92
R5: Age ≥ 47 & WBC < 6.3 & RDW < 43.8 & WBC ≥ 4.6 & Sex(female) & MCHC ≥ 33.4	25.21	74.79
R6: Age ≥ 47 & WBC < 6.3 & RDW < 43.8 & WBC ≥ 4.6 & Sex(female) & MCHC < 33.4	43.22	56.78
R7: Age ≥ 47 & WBC < 6.3 & RDW < 43.8 & WBC ≥ 4.6 & Sex(male) &PDW ≥ 10.3	48.31	51.69
R8: Age ≥ 47 & WBC < 6.3 & RDW < 43.8 & WBC ≥ 4.6 & Sex(male) & PDW < 10.3	92.82	7.18
R9: Age ≥ 47 & WBC < 6.3 & RDW < 43.8 & WBC < 4.6	65.56	34.44
R10: Age ≥ 47 & WBC < 6.3 & RDW ≥ 43.8 & WBC ≥ 4.6 & PLT ≥ 183	58.11	41.89
R11: Age ≥ 47 & WBC < 6.3 & RDW ≥ 43.8 & WBC ≥ 4.6 & PLT < 183	82.28	17.72
R12: Age ≥ 47 & WBC < 6.3 & RDW ≥ 43.8 & WBC < 4.6 & Age ≥ 51	76.03	23.97
R13: Age ≥ 47 & WBC < 6.3 & RDW ≥ 43.8 & WBC < 4.6 & Age < 51	98.93	1.07
R14: Age < 47 & WBC ≥ 6.1 & Age ≥ 40 & RDW < 44.8 & PLT ≥ 284	32.13	67.87
R15: Age < 47 & WBC ≥ 6.1 & Age ≥ 40 & RDW < 44.8 & PLT < 284 & RDW < 41.6	43.36	56.64
R16: Age < 47 & WBC ≥ 6.1 & Age ≥ 40 & RDW < 44.8 & PLT < 284 & RDW ≥ 41.6	62.75	37.25
R17: Age < 47 & WBC ≥ 6.1 & Age ≥ 40 & RDW ≥ 44.8 & MCHC < 31.3 & RDW < 46.2	5.13	94.87
R18: Age < 47 & WBC ≥ 6.1 & Age ≥ 40 & RDW ≥ 44.8 & MCHC < 31.3 & RDW ≥ 46.2	75.22	24.78
R19: Age < 47 & WBC ≥ 6.1 & Age ≥ 40 & RDW ≥ 44.8 & MCHC ≥ 31.3	81.37	18.63
R20: Age < 47 & WBC ≥ 6.1 & Age < 40 & PDW ≥ 14	48.23	51.77
R21: Age < 47 & WBC ≥ 6.1 & Age < 40 & PDW < 14 & RBC ≥ 4.84	66.84	33.16
R22: Age < 47 & WBC ≥ 6.1 & Age < 40 & PDW < 14 & RBC < 4.84	87.06	12.94
R23: Age < 47 & WBC < 6.1 & RDW < 39.4 & HCT ≥ 39.2	51.67	48.33
R24: Age < 47 & WBC < 6.1 & RDW < 39.4 & HCT < 39.2	80.44	19.56
R25: Age < 47 & WBC < 6.1 & RDW ≥ 39.4	83.41	16.59

**Table 4:** Model performance indices of the LR, DT, and BF algorithms for models I and II.

Model II			Model I		
Test (n = 2244)			Test (n = 2250)		
Actual	Predicted count		Actual	Predicted count	
	Diabetic	Non- diabetic		Diabetic	Non- diabetic
**LR Model**					
Diabetic	769	384	Diabetic	761	363
Non-Diabetic	383	708	Non-Diabetic	373	753
Specificity = 64.89%; AUC = 70.76%; Accuracy = 65.81%; Precision = 66.75%			Specificity = 66.87%; AUC = 71.99%; Accuracy = 67.28%; Precision = 67.10%		
**DT Model**					
Diabetic	605	552	Diabetic	792	333
Non-Diabetic	270	817	Non-Diabetic	426	699
Specificity = 75.16%; AUC = 70.01%; Accuracy = 63.36%; Precision = 69.14%			Specificity = 62.13%; AUC = 72.52%; Accuracy = 66.26%; Precision = 65.02%		
**BF Model**					
Diabetic	978	179	Diabetic	1066	59
Non-Diabetic	195	892	Non-Diabetic	139	986
Specificity = 82.06%; AUC = 91.49%; Accuracy = 83.33%; Precision = 83.37%			Specificity = 96.53%; AUC = 99.69%; Accuracy = 97.43%; Precision = 96.59%		

### BF model

**Figure 3:**
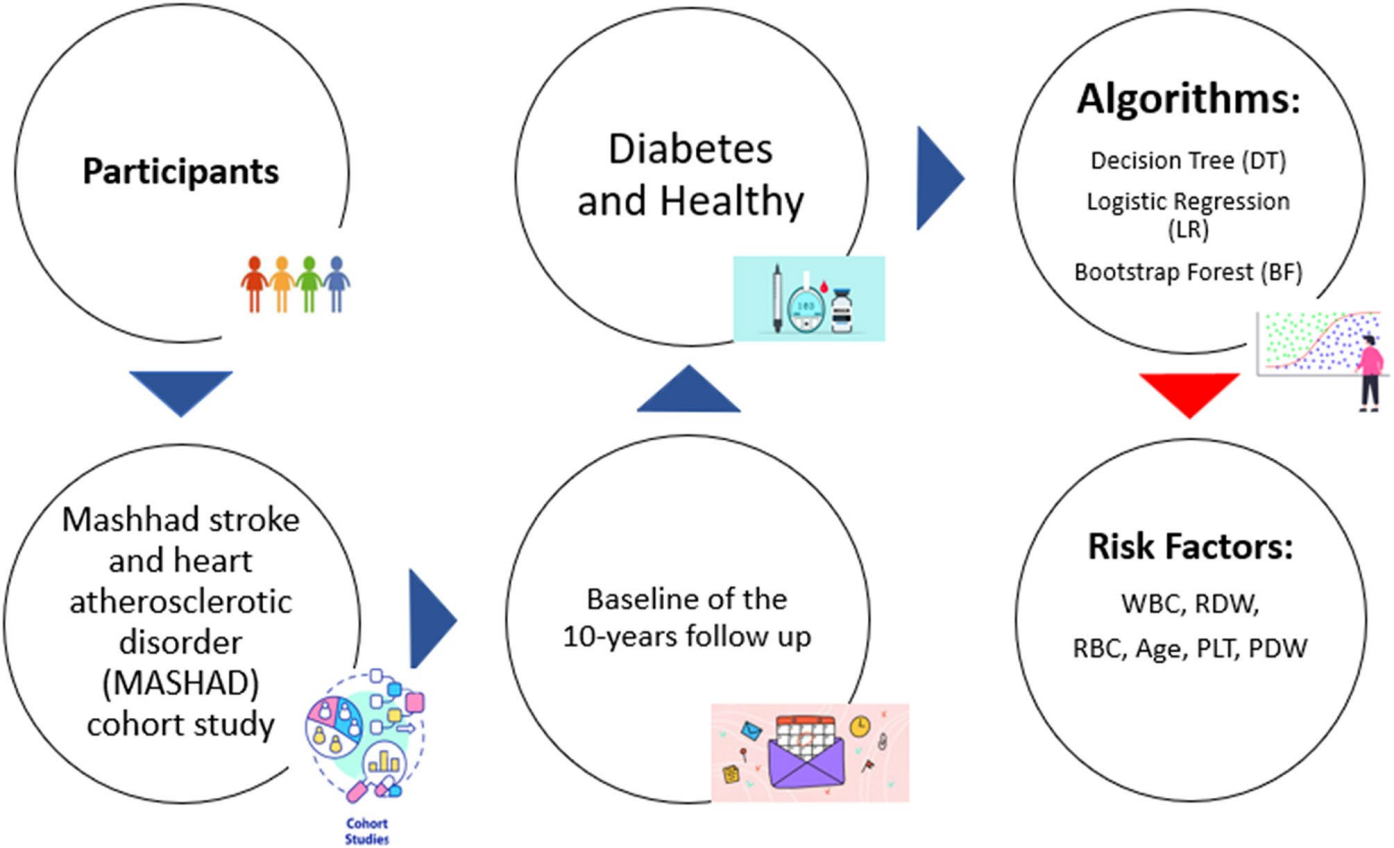
Graphical Abstract.

Finally, for another analysis we used BF for classification the data based on diabetes. The factors included in this BF algorithm are 9 hematological factors for model I and 10 hematological factors for model II used in previous models. Also, in this case, we set the following specifications: Number of Trees in the Forest: 43, Number of Terms Sampled per Split: 2, Training Rows: 6750 for model I and 6734 for model II, Test Rows: 2250 for model I and 2244 for model II, Minimum Splits per Tree: 10, Minimum Size Split: 9. Again for comparison, the confusion matrices of the models I and II are given in Table 4. Moreover, Fig. 2 (e) and (f) show the ROC curves of the models I and II. As shown in Table 4 the accuracy of the models I and II are 83.33 and 97.43 percent. Furthermore, the important variables associated with T2DM based on BF algorithm are given as: Age, WBC, PLT, RBC, RDW, PDW, HCT, MCHC, and Sex in model I and Age, WBC, RBC, HGB, RDW, PDW, PLT, HCT, MCHC, and Sex in model II. As one can observe Age, and WBC were the most significant factors which equal to the obtained results from LR and DT models. We summarize this study in a graphical abstract in Fig. 3.

## Discussion

In this study, a large number of biological and hematological factors like age, WBC, PDW, RDW, RBC, Sex, PLT, MCHC, and HCT had a significant relationship with T2DM. As we mentioned previously, we considered the association of the TyG index with hematological factors because of its positive relationship with T2DM presence. We found that the association of hematological factors with the TyG index was aligned with their results regarding T2DM, except MCHC. Therefore, we will continue the discussion based on the results of the T2DM and hematological factors. The most important and effective factors associated with T2DM presence were found to be age (as the most important and significant factors in the first line of DT) and WBC (as the second factor).

We found that in people over age of 47, the risk of diabetes increased dramatically. In line with our study, one study conducted in western Algeria on a sample of 1852 subjects, get these results with age 50^25^. In another study, the researchers indirectly found that the prevalence of T2DM was higher in middle-aged patients than in younger patients^26^. Contrary to our findings, a study on 307 diabetics showed that age had no significant relationship with the incidence or prevalence of diabetes^1^.

Our findings show that the WBC may be associated with the presence of T2DM. In people with a WBC ≥ 5.4, the prevalence of diabetics was 4 times more than of non-diabetics. Similarly, Lindsay et al. found that high WBC has the protentional to be considered as T2DM after adjusting for age and sex^31^. Another study conducted in 2018 showed that high WBC count, a marker of subclinical inflammation, can be used as an indicator of T2DM due to obesity^32^.

One of the most important difficulties for diabetics is the increased risk of thrombotic events and coagulation problems^33^. Platelets, are the main cellular element of coagulation, and play an important role in this process, and disruption in their number, shape, and activation pathways (measured by PT and MPV criteria) can lead to coagulation problems. The results of our study indicated a direct association between PLT count and the risk of diabetes. Conversely to our findings, the results obtained from a study of 1852 Algerian subjects with 1059 type 2 diabetic patients showed negative effect of PLT on the onset of T2DM^25^ and Some studies just showed that PLT levels are not involved in the development of diabetes pathology^34,35^. The association between PLT and MPV and their effects on each other has been investigated and confirmed in other studies, but surprisingly we could not find any significant association between MPV and the incidence of diabetes. Similarly, a number of studies could not find any association^36–39^, but some have found conflicting results with showing positive effects^40–42^.

The association of RDW and diabetes are stated in different studies. G. Smith and et al. found that Low RDW is associated with increased incidence of T2DM^43^ .Nevertheless, a study conducted in China in 2018 shows a direct link between RDW and the incidence of diabetes^44^.

We also found that HCT was negatively associated with the presence of diabetes, and a 2020 study in Northwest Ethiopia confirmed this inverse relationship^45^. But in another study, they could not find a significant link between HCT and diabetes^46^.

We also found that like high WBC, high RBC and MCHC can also increase the risk of diabetes. As shown in the decision tree, it can be inferred that a decrease in RBC, lower than 4.73, can greatly decrease the risk of diabetes.

Similar to our results, a study of 87 bangal T2DM showed a correlation between high MCHC and RDW with T2DM^47^. However, the study carried out in Saudi Arabia on a population with T2DM showed a negative association between diabetes and MCHC^48^. And so, this factor needs to be further investigated to determine its exact link to diabetes.

According to the results of the study, we obtained that for each unit increase in RBC, the odds of having diabetes 1.64 times which indicates a strong effect of red blood cell count on the risk of T2DM. However, very few studies in the world have linked this factor, and most studies have only reported the effect of T2DM on changes in the appearance and properties of red blood cells^49–51^. Even a 2013 study by Zhan-Sheng Wang and Zhan-Chun Song, which examined the relationship between red blood cell count and its effect on microvascular complications in Chinese patients with T2DM, yielded conflicting results. It was found that the proportion of patients with microvascular complications increases with decreasing red blood cell count (p value below 0.001)^52^. Another study in India in 2019 examined the association and role of hematological factors in diabetes mellitus reported that poorly controlled diabetics were more likely to develop anemia^53^.

One of the most important strengths of our study is the large sample size used. The second strength is the wide age range used in the study, which easily includes the three age groups of young, middle-aged, and elderly, and examines this relationship in them. Also, in this study we examined a relatively large number of hematological factors and for some of these factors not many studies have been done globally yet.

One of the limitations of this study is that we did not measure HbA1c in participants of the MASHAD cohort study. Moreover, it would have been much better if we could have enriched the target community in terms of cultural diversity because our study population was adults in the Mashhad cohort who all live in a common geographical area with relatively similar customs and lifestyles. This makes it impossible to generalize the results of this study to the other countries or even the total population of Iran.

The results of this study can help health authorities in early diagnosis and prevention of diabetes by examining only a few simple hematological criteria.

## Conclusion

Our study showed that the BF model showed a better performance for the prediction of T2DM than the DT and LR models. According to our results, it may be concluded that some of the hematological factors could be valuable tool in the prediction of T2DM such as WBC, PDW, RDW, RBC, PLT, MCHC, and HCT. Among these hematological factors, WBC had the most significant role in the prediction of T2DM. Our findings indicates that hematological factors can be of value for using in the health care setting to predict the T2DM, as they are cost-effective, accessible, and simple markers.

## Supplementary Information

Supplementary Information.

## Data Availability

Data sharing is not applicable to this article as no new data were created in this study. Further enquiries can be directed to the corresponding author.

## References

[1] Demirtas L, Degirmenci H, Akbas EM, Ozcicek A, Timuroglu A, Gurel A (2015). Association of hematological indicies with diabetes, impaired glucose regulation and microvascular complications of diabetes. Int. J. Clin. Exp. Med..

[2] Xu G, Liu B, Sun Y, Du Y, Snetselaar LG, Hu FB (2018). Prevalence of diagnosed type 1 and type 2 diabetes among US adults in 2016 and 2017: Population based study. BMJ.

[3] LeRoith D, Biessels GJ, Braithwaite SS, Casanueva FF, Draznin B, Halter JB (2019). Treatment of diabetes in older adults: An endocrine society* clinical practice guideline. J. Clin. Endocrinol. Metab..

[4] Saeedi P, Petersohn I, Salpea P, Malanda B, Karuranga S, Unwin N (2019). Global and regional diabetes prevalence estimates for 2019 and projections for 2030 and 2045: Results from the international diabetes federation diabetes atlas. Diabetes Res. Clin. Pract..

[5] Najafipour, H., Farjami, M., Sanjari, M., Amirzadeh, R., Shadkam Farokhi, M., Mirzazadeh, A. Prevalence and incidence rate of diabetes, pre-diabetes, uncontrolled diabetes, and their predictors in the adult population in southeastern Iran: Findings From KERCADR Study. *Front. Public Health*.9 (2021). 10.3389/fpubh.2021.611652 PMC8591105 34790639

[6] Jones RL, Peterson CM (1981). Hematologic alterations in diabetes mellitus. Am. J. Med..

[7] Arkew M, Yemane T, Mengistu Y, Gemechu K, Tesfaye G (2021). Hematological parameters of type 2 diabetic adult patients at debre berhan referral hospital, Northeast Ethiopia: A comparative cross-sectional study. PLoS ONE.

[8] Engström G, Smith JG, Persson M, Nilsson PM, Melander O, Hedblad B (2014). Red cell distribution width, haemoglobin A1c and incidence of diabetes mellitus. J. Intern. Med..

[9] Milosevic D, Panin VL (2019). Relationship between hematological parameters and glycemic control in type 2 diabetes mellitus patients. J. Med. Biochem..

[10] Ghayour-Mobarhan M, Moohebati M, Esmaily H, Ebrahimi M, Parizadeh SMR, Heidari-Bakavoli AR (2015). Mashhad stroke and heart atherosclerotic disorder (MASHAD) study: Design, baseline characteristics and 10-year cardiovascular risk estimation. Int. J. Public Health.

[11] Hameed EK (2019). TyG index a promising biomarker for glycemic control in type 2 diabetes mellitus. Diabetes Metab Syndr..

[12] Lusa L (2013). Improved shrunken centroid classifiers for high-dimensional class-imbalanced data. BMC bioinformatics.

[13] David W. Hosmer Jr. SL, Rodney X. Sturdivant. Applied Logistic Regression. 3rd, editor. Hoboken, New Jersey: John Wiley & Sons Inc. (2013).

[14] Saberi-Karimian M, Khorasanchi Z, Ghazizadeh H, Tayefi M, Saffar S, Ferns GA (2021). Potential value and impact of data mining and machine learning in clinical diagnostics. Crit. Rev. Clin. Lab. Sci..

[15] Mohammadi M, Mansoori A (2018). A projection neural network for identifying copy number variants. IEEE J. Biomed. Health Inform..

[16] Zhong, Y., editor The analysis of cases based on decision tree.In *2016 7th IEEE international conference on software engineering and service science (ICSESS)* IEEE (2016).

[17] Aghasizadeh M, Samadi S, Sahebkar A, Miri‐Moghaddam E, Esmaily H, Souktanloo M, et al. Serum HDL cholesterol uptake capacity in subjects from the MASHAD cohort study: Its value in determining the risk of cardiovascular endpoints. *J. Clin. Lab. Anal.*:e23770 (2021). 10.1002/jcla.23770 PMC8183926 34028874

[18] Saberi‐Karimian, M., Safarian‐Bana, H., Mohammadzadeh, E., Kazemi, T., Mansoori, A., Ghazizadeh, H., et al. A pilot study of the effects of crocin on high‐density lipoprotein cholesterol uptake capacity in patients with metabolic syndrome: A randomized clinical trial. *BioFactors.* (2021). 10.1002/biof.1783 34609029

[19] Saberi-Karimian M, Mansoori A, Bajgiran MM, Hosseini ZS, Kiyoumarsioskouei A, Rad ES, Zo MM, Khorasani NY, Poudineh M, Ghazizadeh S, Ferns G, Esmaily H, Ghayour-Mobarhan M (2023). Data mining approaches for type 2 diabetes mellitus prediction using anthropometric measurements. J. Clin. Lab. Anal..

[20] Hooley JM, Teasdale JD (1989). Predictors of relapse in unipolar depressives: Expressed emotion, marital distress, and perceived criticism. J. Abnorm. Psychol..

[21] Mohammadi, F., Pourzamani, H., Karimi, H., Mohammadi, M., Mohammadi, M., Ardalan, N., et al. Artificial neural network and logistic regression modelling to characterize COVID-19 infected patients in local areas of Iran. *Biomed. J*. (2021). 10.1016/j.bj.2021.02.006 PMC7905378 34127421

[22] Al-Azzam N, Elsalem L, Gombedza F (2020). A cross-sectional study to determine factors affecting dental and medical students’ preference for virtual learning during the COVID-19 outbreak. Heliyon.

[23] Chamroonkiadtikun P, Ananchaisarp T, Wanichanon W (2020). The triglyceride-glucose index, a predictor of type 2 diabetes development: A retrospective cohort study. Prim. Care Diabetes.

[24] Park B, Lee HS, Lee Y-J (2021). Triglyceride glucose (TyG) index as a predictor of incident type 2 diabetes among nonobese adults: A 12-year longitudinal study of the korean genome and epidemiology study cohort. Transl. Res..

[25] Kachekouche Y, Dali-Sahi M, Benmansour D, Dennouni-Medjati N (2018). Hematological profile associated with type 2 diabetes mellitus. Diabetes Metab. Syndr..

[26] Feldman-Billard S, Sedira N, Boelle P-Y, Poisson F, Héron E (2013). High prevalence of undiagnosed diabetes and high risk for diabetes using HbA1c criteria in middle-aged patients undergoing cataract surgery. Diabetes Metab..

[27] Lee J-W, Lim N-K, Park H-Y (2018). The product of fasting plasma glucose and triglycerides improves risk prediction of type 2 diabetes in middle-aged Koreans. BMC Endocr. Disord..

[28] Navarro-González D, Sánchez-Íñigo L, Pastrana-Delgado J, Fernández-Montero A, Martinez JA (2016). Triglyceride–glucose index (TyG index) in comparison with fasting plasma glucose improved diabetes prediction in patients with normal fasting glucose: The vascular-metabolic CUN cohort. Prev. Med..

[29] Bennett C, Guo M, Dharmage S (2007). HbA1c as a screening tool for detection of type 2 diabetes: A systematic review. Diabet. Med..

[30] Selvi NMK, Nandhini S, Sakthivadivel V, Lokesh S, Srinivasan AR, Sumathi S (2021). Association of triglyceride-glucose index (TyG index) with HbA1c and insulin resistance in type 2 diabetes mellitus. Maedica.

[31] Lindsay R (2002). High white blood cell count is associated with a worsening of insulin sensitivity and predicts the development o. Diabetes.

[32] Gu Y, Hu K, Huang Y, Zhang Q, Liu L, Meng G (2018). White blood cells count as an indicator to identify whether obesity leads to increased risk of type 2 diabetes. Diabetes Res. Clin. Pract..

[33] Kim JH, Bae HY, Kim SY (2013). Clinical marker of platelet hyperreactivity in diabetes mellitus. Diabetes Metab. J..

[34] Kodiatte TA, Manikyam UK, Rao SB, Jagadish TM, Reddy M, Lingaiah HKM (2012). Mean platelet volume in type 2 diabetes mellitus. J. Lab. Physicians..

[35] Zhang M, Zhang Y, Li C, He L (2015). Association between red blood cell distribution and renal function in patients with untreated type 2 diabetes mellitus. Ren. Fail..

[36] Ozder A, Eker HH (2014). Investigation of mean platelet volume in patients with type 2 diabetes mellitus and in subjects with impaired fasting glucose: A cost-effective tool in primary health care?. Int. J. Clin. Exp. Med..

[37] Jabeen F, Fawwad A, Rizvi HA, Alvi F (2013). Role of platelet indices, glycemic control and hs-CRP in pathogenesis of vascular complications in type-2 diabetic patients. Pak. J. Med. Sci..

[38] Zaccardi F, Rocca B, Pitocco D, Tanese L, Rizzi A, Ghirlanda G (2015). Platelet mean volume, distribution width, and count in type 2 diabetes, impaired fasting glucose, and metabolic syndrome: A meta-analysis. Diabetes Metab. Res. Rev..

[39] Erdoğan S, Özdemir Ö, Doğan HO, Sezer S, Atalay CR, Yilmaz FM (2014). Liver enzymes, mean platelet volume, and red cell distribution width in gestational diabetes. Turkish J. Med. Sci..

[40] Lippi G, Salvagno GL, Nouvenne A, Meschi T, Borghi L, Targher G (2015). The mean platelet volume is significantly associated with higher glycated hemoglobin in a large population of unselected outpatients. Prim. Care Diabetes.

[41] Akinsegun, A., Olusola, D.A., Sarah, J.-O., Olajumoke, O., Adewumi, A., Majeed, O., et al. Mean platelet volume and platelet counts in type 2 diabetes: mellitus on treatment and non-diabetic mellitus controls in Lagos, Nigeria. Pan Afr. Med. J. 18 (2014). 10.11604/pamj.2014.18.42.3651 PMC4215377 25368731

[42] Hekimsoy Z, Payzin B, Örnek T, Kandoğan G (2004). Mean platelet volume in Type 2 diabetic patients. J. Diabetes Complicat..

[43] Engström G, Smith J, Persson M, Nilsson P, Melander O, Hedblad B (2014). Red cell distribution width, haemoglobin A 1c and incidence of diabetes mellitus. J. Intern. Med..

[44] Zhang J, Zhang R, Wang Y, Li H, Han Q, Wu Y (2018). The association between the red cell distribution width and diabetic nephropathy in patients with type-2 diabetes mellitus. Ren. Fail..

[45] Adane T, Getaneh Z, Asrie F (2020). Red blood cell parameters and their correlation with renal function tests among diabetes mellitus patients: A comparative cross-sectional study. Diabetes Metab. Syndr. Obes. Targets Ther..

[46] Berria R, Glass L, Mahankali A, Miyazaki Y, Monroy A, De Filippis E (2007). Reduction in hematocrit and hemoglobin following pioglitazone treatment is not hemodilutional in type II diabetes mellitus. Clin. Pharmacol. Ther..

[47] Jaman MS, Rahman MS, Swarna RR, Mahato J, Miah MM, Ayshasiddeka M (2018). Diabetes and red blood cell parameters. Ann. Clin. Endocrinol. Metabol..

[48] Waggiallah H, Alzohairy M (2011). The effect of oxidative stress on human red cells glutathione peroxidase, glutathione reductase level, and prevalence of anemia among diabetics. N. Am. J. Med. Sci..

[49] Rand PW, Norton JM, Barker ND, Richards AL, Lacombe EH, Pirone LA (1981). Effects of diabetes mellitus on red cell properties. Clin. Hemorheol. Microcirc..

[50] Moon J, Kim J, Park I, Lee J, Kim H, Lee J (2016). Impaired RBC deformability is associated with diabetic retinopathy in patients with type 2 diabetes. Diabetes Metab..

[51] Vahalkar GS, Haldankar VA (2008). RBC membrane composition in insulin dependent diabetes mellitus in context of oxidative stress. Indian J. Clin. Biochem..

[52] Wang Z-S, Song Z-C, Bai J-H, Li F, Wu T, Qi J (2013). Red blood cell count as an indicator of microvascular complications in Chinese patients with type 2 diabetes mellitus. Vasc. Health Risk Manag..

[53] Farooqui R, Afsar N, Afroze IA (2019). Role and significance of hematological parameters in diabetes mellitus. Annal. Pathol. Lab. Med..

