# Supplementary material for: Prediction of type 2 diabetes mellitus using hematological factors based on machine learning approaches: a cohort study analysis
Source: Sci Rep. 2023 Jan 12;13:663. doi: 10.1038/s41598-022-27340-2 (PMC9837189; doi:10.1038/s41598-022-27340-2)
Supplement: Supplementary file 1 — Supplementary Information. [file 41598_2022_27340_MOESM1_ESM.docx]

**Figure S1. Graphical representation of the classification tree for model I.**

**Figure S2. Graphical representation of the classification tree for model II.**
